# Detection of fungal and bacterial contamination of hazelnut and determination of aflatoxin B by HPLC method in Isfahan, Iran

**DOI:** 10.18502/cmm.7.4.8404

**Published:** 2021-12

**Authors:** Elham Saffari, Mahboobeh Madani, Vajihe Karbasizade, Pegah Shakib

**Affiliations:** 1 Department of Microbiology, Falavarjan Branch, Islamic Azad University, Isfahan, Iran; 2 Department of Microbiology, School of Medicine, Isfahan University of Medical Sciences, Isfahan, Iran; 3 Razi Herbal Medicines Research Center, Lorestan University of Medical Sciences, Khorramabad, Iran

**Keywords:** Aflatoxin, HPLC, Iran, Nuts

## Abstract

**Background and Purpose:**

Due to the fact that fungal species, such as *Aspergillus flavus* and *Aspergillus parasiticus* produce carcinogenic and mutagenic aflatoxins and have the potential to produce fungal secondary metabolites, fungal contamination should be avoided. This study was conducted using the HPLC method and aimed to examine the fungal contamination of Isfahan hazelnuts in order to identify the presence of Aflatoxins.

**Materials and Methods:**

In total, 100 samples of hazelnuts were randomly collected from supermarkets in Isfahan. The samples were then cultured on Sabouraud dextrose agar media and analyzed to determine fungal contaminations. The aflatoxin analysis was carried out using the HPLC method.

**Results:**

It was discovered that nine genera of fungi, namely *Aspergillus*, *Penicillium*, *Rhizopus*, *Ulocladium*, *Alternaria*, *Drechselera*, *Trichothecium*, *Scopulariopsis*, and *Mucor* were identified in 78% of the samples. Samples contaminated with *Aspergillus flavus* (22 samples) were studied to determine the presence of aflatoxin. The results showed that 16 (72.72%) of the samples were contaminated with AFB1, AFB2, and AFG2 and the mean concentrations were 0.926, 0.563, and 0.155 ng/g, respectively.

**Conclusion:**

Some parameters that affect mycotoxin production are temperature, food substrate, the strain of the mold, and other environmental factors. Due to the toxigenic quality of some of these fungi and their hazard to human health, it is crucial that fungal contamination and aflatoxin identification tests are carried out before certain products are made available to the mass market.

## Introduction

Hazelnuts are one of the products that are exposed to bacterial and fungal contamination and this is a major problem for farmers in many countries because germs are responsible for a wide range of harmful effects [ 1 , 2 ]. Hazelnuts are rich in protein, minerals, unsaturated fats, and vitamins, hence they may be contaminated by microorganisms, such as bacteria and fungi in various stages on the tree, at harvest time and during storage. Bacteria, such as coliforms, *Staphylococcus aureus* (*S. aureus*), and fungi can cause microbial contamination of the hazelnut [ 3 , 4 ]. 

Mycotoxins are produced by a taxonomically wide variety of filamentous fungi and a diverse group of compounds. Many mycotoxin diseases are associated with various species of fungal genera and their secondary metabolites [ 5 ]. The adverse effects of fungal products have caused mass poisoning in both humans and farm animals in many countries. *Aspergillus* spp, especially *Aspergillus flavus* (*A. flavus*) and *A. parasiticus* species, naturally produce mycotoxins, such as aflatoxins (AFs) [ 6 ]. These AFs are severely toxic, immunosuppressive, mutagenic, teratogenic, and carcinogenic compounds [ 7 ]. It is no surprise that the International Agency for Research on Cancer classified these four aflatoxins (AFB1, AFB2, AFG1, and AFG2, respectively) as group 1 carcinogens and aflatoxin B1 (AFB1) as the most significant carcinogenic agent. [ 8 , 9 ].

The Food and Agricultural Organization estimates that each year, 25-50% of crops are contaminated with mycotoxins globally [ 10 ]. Hazelnuts (*Corylus* spp), also known as filberts, are a major commercial crop in many countries that are prone to mold contamination during all stages of their production: growth, harvesting, processing, and storage [ 11 ]. This can possibly lead to the production of secondary toxic metabolites, known as mycotoxins. Therefore, one of the main concerns of health officials is the control of fungal toxins. In this regard, several studies have been conducted to control fungal toxins in various agricultural products, including almonds, pistachios, and other products [ 12 - 14 ]. Therefore, the purpose of this study was to detect fungal and bacterial contamination of Isfahan hazelnut and determine aflatoxin B by the HPLC method in Isfahan, Iran.

## Materials and Methods

### Sample collection

In total, 100 samples of hazelnut were randomly selected from supermarkets in Isfahan, and sampling was done randomly. Samples were cultured as sterile (to isolate fungi) and non-sterile (to isolate bacteria). To isolate fungi, the samples were soaked in a sodium hypochlorite solution (5%) for 3 min, washed in sterile distilled water, and placed between sterile sheets (surface disinfection of samples is a standard way to remove surface contamination of food for research to remove airborne fungal spores, which are usually present in large numbers, from the food surface and cause misleading results during the test. Note that in this research, this issue was observed [ 15 ].

Subsequently, the samples of hazelnut were cultured on Sabouraud Dextrose Agar and then incubated at 25 oC for 4 days. Fungal colonies around the grains were purified and maintained on slant Potato Dextrose Agar for the purpose of identification trials. The isolated fungi were then evaluated using mycological methods[ 16 ]. 

For isolation of bacterial isolates, the non-sterile sample was cultured on Nutrient Agar (NA) and incubated at 37 oC. In addition, the number of bacterial colonies grown on NA on each plate was counted and the average count was calculated. To examine the average colony count, the number of colonies in three plates related to each dilution was counted and the average count was calculated and then the bacterial count in the prototype (CFU/g) was calculated using the following equation:

Dilution factor×Cultivated sample size/mean colony count in three plates=number of bacteria in the prototype.

After counting the grown bacterial colonies and gram staining, identification of microbial isolates by standard microbial and biochemical tests including catalase, coagulase, DNA hydrolysis, novobiocin susceptibility, TSI, indole, methyl red (MR), Voges-Proskauer (VP), urease, motility, use of citrate, and fermentation of sugars were performed.

### Sample Preparation and Clean up

In order to reduce the sampling error in AF analysis, the water slurry of hazelnut samples was prepared. To do so, 1.5 L of water was added per kilogram of hazelnuts (15 L for each 10 kg subsample). Using a slurry machine, the mixture was ground for 15 minutes and was turned into a slurry. Finally, 125 g of the slurry was taken as a test portion for the purpose of the analysis.

The samples were analyzed using the HPLC model (the AOAC official method 999. 07) although some minor adjustments had to be made. The hazelnut slurries were extracted using methanol/water/ hexane (300 mL/75 mL/100 mL). The extract was then diluted with water and passed through the glass microfiber filters. To clean up the samples, the following procedure was carried out using the Alfa test IACs method. first, 10 mL of phosphate saline buffer (PBS) was passed through the IAC. Then, 75 mL of the filtrate (10 mL extract+65 mL PBS) was passed through the IAC at the flow rate of ca. 1 drop/s.; the column was washed with 15 mL PBS and dried using a small vacuum, and finally, AFs were eluted with methanol. To do so, 0.5 mL was introduced into the column passed through due to gravity. After 1 min, the second portion of 0.75 mL methanol was injected into the column. The eluate was diluted with water and analyzed by HPLC [ 17 ].

### AF Standards

The AF standards (Rodricks 1973) were prepared from Sigma Chemical Company, St. Louis, MO. Standard solutions of individual AFs were made to prepare mixed working standards for HPLC analysis. The concentration of each standard solution was measured using the UV spectrophotometer [ 17 ].

### Analysis of AF using HPLC

In order to quantify the AFs, the reverse-phase HPLC and fluorescence detector with Post Column Derivatization Chamber (PCDC) involving bromination were used. By adding potassium bromide to the mobile phase and using a Kobra cell, the PCDC was achieved. The AF elute was thinned with water and 100 mL of the thinned solution was injected into HPLC. The analytical column used was C18, 5 mm, 250 mm, and 4.6 mm i.d. The mobile phase was water: methanol: acetonitrile (54:29:17, v/v/v) with a flow rate of 1 mL/min. The fluorescence detector operation was performed at an excitation wavelength of 365 nm and an emission wavelength of 435 nm.

A 5-point calibration curve was built daily for every AF, including AFB1, AFB2, AFG1, and AFG2. The curves were analyzed for linearity and were used for the quantification of AF in the hazelnut samples. The AFG2 was eluted first followed by AFG1, AFB2, and AFB1 [ 17 ].

### Statistical analysis

Data were analyzed by SPSS 18 software and the Kruskal-Wallis test was used to compare the levels of four aflatoxins (*P*<0.001).

## Results

### Bacterial and fungal isolation

From 100 samples of hazelnuts (sterile and non-sterile) collected from Isfahan, three bacterial genera, including *Bacillus megaterium* (*B. megaterium*), *B. licheniformis*, *B. circulans*, *B. subtilis*, *B. cereus*, *Escherichia coli* (*E. coli*), and *S. aureus* were isolated. The highest frequency of isolated bacteria was related to *Bacillus*. Moreover, 60% of non-sterile specimens were infected with *Bacillus*, the highest frequency of *Bacillus* was related to *B. megaterium* (23%), followed by *B. licheniformis* (16%), *S. aureus* (17%), and *E. coli* (5%). Therefore, 58% of non-sterile samples had bacterial contamination. In addition, 26% of the sterilized samples were infected with *Bacillus* and the highest frequency of *Bacillus* was related to *B. megaterium* (8%), followed by *B. licheniformis* (7%) and *S. aureus* (3%). Therefore, 27% of the sterilized samples had bacterial contamination.

According to the results, nine genera of fungi, including *Aspergillus*, *Penicillium*, *Rhizopus*, *Ulocladium*, *Alternaria*, *Drechselera*, *Trichothecium*, *Scopulariopsis*, Mucor, were identified in 78% of the samples. Isolated *Aspergillus* were *A. niger* (66%), *A. flavus* (22%), *A. fumigatus* (21%) and *A. terreus* (4%). The predominantly isolated fungus was *A. niger*, followed by *Penicillium*, *Rhizopus*, and *Alternaria*, respectively.

### Aflatoxin analysis using the HPLC method

Contaminated samples with *A. flavus* (22 samples in total) were tested for aflatoxin using the HPLC method. The results proved that 16 (77.72%) of the samples were tainted with AFB1, AFB2, and AFG2 and the mean concentrations were 0.926, 0.563, and 0.155 ng/g, respectively.

The amount of total aflatoxin in the above 16 samples was within the allowable standard of Iran (maximum 15 ng g^−1^ = ppb) and in none of the samples, the amount of aflatoxin was more than the allowable limit. The highest concentration of total aflatoxin was 4.18 ppb and the highest amount of aflatoxin B1 was 1.98 ppb.

The concentration of B1, B2, G1, and G2 aflatoxins was detected by HPLC in 22 samples contaminated with A. flavus. In total, 16 (72.7%) out of the 22 samples contaminated with *A. flavus* were contaminated with aflatoxin (Table 1). As shown in Table 2, 68.18% of samples were contaminated with AFB1 with mean concentrations of 0.926 ng g^−1^; 27.27% of samples were contaminated with AFB2 with mean concentrations of 0.563 ng g^−1^. Moreover, 36.36% of the samples were contaminated with AFG2 with mean concentrations of 0.155 ng g^−1^. In addition, AFG1 was not detected in this study. The occurrence of aflatoxin in samples was lower than the 15 ng g^-1^ by Codex Alimentarius [ 18 ]. Therefore, the results shown in Table 1 are within the acceptablrange.

**Table 1:** Concentration of aflatoxins B1, B2, G1, G2, and total aflatoxin

Number	B1	B2	G1	G2	Total aflatoxin
1	0/7	ND	ND	ND	0/7
2	ND	ND	ND	ND	ND
3	0/7	ND	ND	ND	0/7
4	0/7	0/15	ND	ND	0/85
5	0/85	0/17	ND	0/13	1/15
6	0/7	ND	ND	ND	0/7
7	ND	ND	ND	0/15	0/15
8	ND	ND	ND	ND	ND
9	0/7	ND	ND	0/2	0/9
10	1/85	0/5	ND	ND	2/35
11	0/7	ND	ND	0/15	0/85
12	ND	ND	ND	ND	ND
13	0/7	0/15	ND	ND	0/85
14	0/7	ND	ND	0/15	0/85
15	0/98	0/21	ND	0/14	1/33
16	ND	ND	ND	ND	ND
17	ND	ND	ND	ND	ND
18	0/7	ND	ND	ND	0/7
19	0/7	ND	ND	0/15	0/85
20	1/98	2/2	ND	ND	4/18
21	ND	ND	ND	ND	ND
22	1/23	ND	ND	0/17	1/4

* ND: Not Detected 0.06 (ppb)>

**Table 2:** Prevalence of aflatoxins in 22 samples analyzed by HPLC

Type of aflatoxin	Positive (%)	Maximum (ppb)	Minimum (ppb)	Mean	Recovery
B1	68/18	1/98	ND	0/926	76
B2	27/27	2/2	ND	0/563	
G1	0	ND	ND	0	78
G2	36/36	0/17	ND	0/155	82/15
Total	72/72	4/18	ND	1/156	84/08

## Discussion

Nuts are among the agricultural products prone to microbial contamination and a breeding ground for fungi and bacteria. A number of fungi have the ability to produce mycotoxins, which seriously threaten human and animal health. Sometimes bacteria enter the food and the toxins it secretes, such as the exotoxin produced by *S. aureus*, *Clostridium botulinum* (*C. botulinum*), *C. perfringens*, and *C. valencia*, poison the consumer.

In this study, *B. licheniformis* was isolated in 16% and 7% of non-sterile and sterile samples, and *B. megaterium* was isolated in 23% and 8% of non-sterile and sterile samples, respectively. The reason for the high frequency of infection with *Bacillus* species is due to the production of spores in these genera and their resistance to drought and unfavorable conditions. In Azizkhani et al., in cashew nut, peanut, and hazelnut, no coliforms were isolated and *Staphylococcus aureus* was found in 25% of cashew nut, 33% of peanut, and 6.5% of hazelnut. In addition, the results showed that the total number of bacteria in the samples was within the standard range [ 11 ]. 

In this study, 78% of hazelnut samples were infected with nine fungal genera. The highest infection rate was related to *Aspergillus* and the lowest was related to *Drechselera*. Susel et al. studied a large number of samples taken from different countries in South America and reported that the hot and humid climate of these countries provides favorable conditions for fungal growth and aflatoxin production [ 19 ]. In a study conducted by Hedayati et al., peanut and pistachio seeds were studied in Sari in terms of fungal infection. Based on their results, contamination of samples with fungal species was about 70%. In addition, *Aspergillus* is the most abundant reported genus (70.5%). 

*A. flavus*, 2.5% *A.niger*, and 14.4% of other species of *Aspergillus* were isolated from 58.6% of the samples. Other fungi isolated from peanuts in this study were *Penicillium* 7.2%, *Cladosporium* 10.9%, Yeast 11.2%, and *Rhodotorola* 0.2% [ 20 ]. Based on the comparison of the results of our study and the aforementioned studies, it was found that despite the difference in climate type of our study area (Isfahan, Iran), which is a dry region with tropical and subtropical regions, such as Mazandaran, *Aspergillus* was the most frequently isolated species.

Kabak in Turkey analyzed 300 samples of hazelnuts for aflatoxin incidence using the HPLC-FLD technique. The results showed that no aflatoxin was found in hazelnut shells, while six samples of raw hazelnut kernels (12%) contained aflatoxin from 0.09 to 11.3 μg kg^−1^, and five samples of roasted hazelnut kernels (8.3%) contained aflatoxin from 0.17 to 11.2 μg kg^−1^[ 21 ]. 

In the present study, the amount of aflatoxin in samples infected with *A. flavus* was evaluated by high-performance liquid chromatography (HPLC) and the results showed the amount of aflatoxins B1, B2, and G2 in the Iranian limit (Maximum 15 ppb) and in none of the samples, the amount of aflatoxin was more than the allowable level. The highest concentration of total aflatoxin was 4.18 ppb and the highest amount of aflatoxin B1 was 1.98 ppb.

In China, Wang et al. measured aflatoxin levels by HPLC in 23.08% of peanut samples with an average level of 0.82 µg/kg and a maximum level of 28.39/g/kg. Aflatoxin B1 in nine samples of peanuts (13.85%) with an average of 0.4 g/kg, aflatoxin B2 in five samples (7.69%) with an average of 0.1/g/kg, and aflatoxin G1 in four samples (6.15) %) with an average of 0.28/g/kg, and in only three samples (4.62%) aflatoxin G2 with an average of 0.04/g/kg were found [ 22 ]. In the present study, total aflatoxin with an average of 1.156 µg/kg, aflatoxin B1 with an average of 0.926/g/kg, aflatoxin B2 with an average of 0.563 kg/kg, and aflatoxin G2 with an average of 0.155/g/kg were evaluated by HPLC.

Out of 22 samples infected with *A. flavus*, 16 samples contained aflatoxins B1, G2, and B2, and the amount of aflatoxin G2 in all samples was 0.0. The genus *Aspergillus* had the highest frequency and among the species of *Aspergillus*, *A. niger* had the highest frequency. The maximum levels of aflatoxin B1 and total in these samples were 1.98 and 4.18 μg/kg, respectively, which was acceptable in Iran.

Aflatoxins are carcinogenic metabolites and teratogens of *A. flavus* and *A. parasiticus*, they are present as vital contaminants in food, especially in peanuts, corn, and other crops found in tropical countries. Aflatoxins cause various adverse toxic effects in humans and they have consequences, such as hepatocellular carcinoma and rapid death [ 23 ]; therefore, due to aflatoxin in different foods, its isolation can be of particular importance.

## Conclusion

This study provided valuable information on fungal and aflatoxin contamination of hazelnuts in Isfahan (arid region of Iran), as the presence of AF in foods, including nuts, can serve as a warning for further investigations to provide measures to prevent aflatoxin contamination of plants.

## Acknowledgement

This article is derived from a thesis submitted for partial fulfillment of the Master's Degree to the Islamic Azad University, Falavarjan branch. The authors would like to thank the experts of Islamic Azad University, Falavarjan branch, Isfahan, Iran.

## Authors’ contribution

The present study was designed in collaboration with E. S.‚ M. M.‚ V. K., and P. S. After performing the necessary experiments to collect data, they were analyzed, and then all authors participated in the preparation of the first version and confirmed the final version.

## Conflict of Interest

The authors have no conflict of interest.

## Financial disclosure

None Declared.
